# Colocutaneous Fistula after Percutaneous Endoscopic Gastrostomy (PEG) Tube Insertion

**DOI:** 10.5811/cpcem.2020.7.48335

**Published:** 2020-10-05

**Authors:** Matthew Warner, Muhammad Durrani

**Affiliations:** Inspira Medical Center, Department of Emergency Medicine, Vineland, New Jersey

**Keywords:** Colocutaneous fistula, percutaneous endoscopic gastrostomy, parenteral nutrition

## Abstract

**Case Presentation:**

A 48-year-old-female presented to the emergency department with dislodgement of her percutaneous endoscopic gastrostomy (PEG) tube, necessitating bedside replacement. Replacement was done without difficulty and gastrografin radiography was obtained to confirm positioning. Radiography revealed contrast filling the colon at the splenic flexure and proximal descending colon suggestive of colocutaneous fistula formation.

**Discussion:**

The patient required hospitalization with surgical consultation, initiation of parenteral nutrition, and conservative management of the fistula with surgical replacement of the PEG tube. Although rare, it is paramount for the emergency physician to be aware of this complication when undertaking bedside replacement of PEG tubes.

## CASE PRESENTATION

A 48-year-old female with past medical history of failure to thrive requiring percutaneous endoscopic gastrostomy (PEG) tube placement presented to the emergency department (ED) with PEG tube dislodgement. Her PEG tube was initially placed in 2009, with surgical replacements in 2012 and 2018. Examination revealed a soft, nontender, non-distended abdomen with an open gastrostomy tract. ED course included placement of a new gastrostomy tube into the existing tract and confirmatory gastrografin radiography (Image).

## DISCUSSION

The confirmatory radiography revealed contrast filling the colon at the splenic flexure and proximal descending colon suggestive of colocutaneous fistula formation, without peritoneal extravasation. This required hospitalization with surgical consultation for removal of the misplaced PEG tube, initiation of parenteral nutrition, intravenous antibiotics, and surgical reinsertion of the PEG tube after conservative management of the colocutaneous fistula. Colocutaneous fistulas are a rare complication of PEG tube insertion with incidence rates of 0.5–3%.^1^ Fistula formation is mediated by PEG tube penetration of interposed colon between the stomach and abdominal wall during the initial insertion.^1^–^5^ Fistula formation occurs at the time of initial insertion but symptoms manifest after reinsertion of the PEG tube fails to completely pass the tube through the interposed colon to enter the stomach.^2^,^3^ Risk factors include adhesions from previous laparotomy, postural and spinal abnormalities, and high-riding transverse colon.^1^ Symptoms include sudden onset diarrhea after PEG tube feeds, visualization of undigested feeding formula, and feculent vomiting with retrograde passage of material from the colon.^1^ Upper endoscopy with water-soluble contrast is the diagnostic modality of choice.^1^–^5^ Treatment ranges from conservative management aimed at decreasing fistula output and allowing for spontaneous closure after infection control, nutritional optimization and establishing wound care to surgical repair. Since emergency physicians change a large number of PEG tubes, recognition and awareness of this rare complication is important to clinical practice.

CPC-EM Capsule

What do we already know about this clinical entity?

*Colocutaneous fistulas are a rare complication of percutaneous endoscopic gastrostomy (PEG) tube insertion mediated by PEG tube penetration of interposed colon between the stomach and abdominal wall*.

What is the major impact of the image(s)?

*Contrast filling the splenic flexure and proximal descending colon suggests colocutaneous fistula formation, requiring prompt recognition and surgical consultation*.

How might this improve emergency medicine practice?

*Emergency physicians change a large number of PEG tubes; therefore, recognition and awareness of this rare complication is important to clinical practice*.

## Figures and Tables

**Image:**
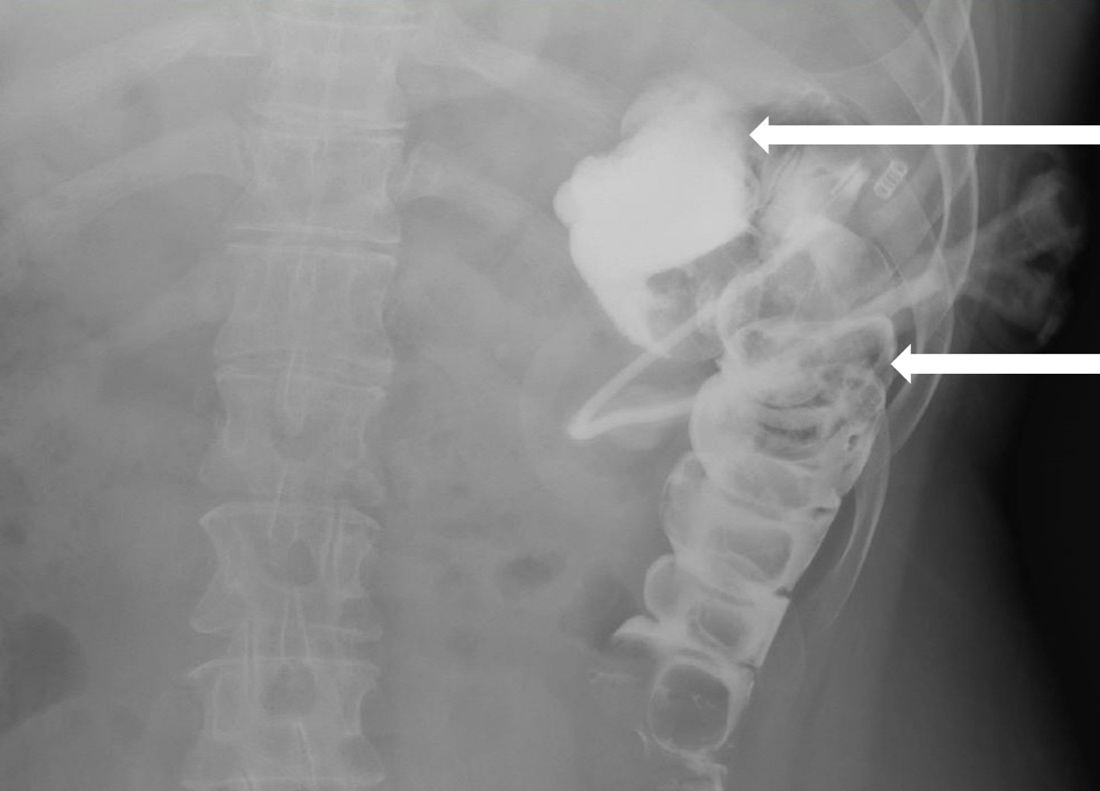
Gastrografin radiography showing percutaneous endoscopic gastrostomy tube in the colon with contrast filling colon at the splenic flexure (top arrow) and proximal descending colon (bottom arrow) suggestive of colocutaneous fistula formation.
